# Multiple teams, multiple projects, multiple groups at the intersection of (multiple) research fields: A bibliometric study

**DOI:** 10.3389/fpsyg.2023.1027349

**Published:** 2023-02-23

**Authors:** Nicoleta Meslec, Petru Lucian Curseu, Oana C. Fodor, Saša Batistič, Renata Kenda

**Affiliations:** ^1^Department of Organisation Studies, Tilburg University, Tilburg, Netherlands; ^2^Department of Psychology, Babes-Bolyai University, Cluj-Napoca, Romania; ^3^Department of Organisation, Open Universiteit, Heerlen, Netherlands; ^4^Department of Human Resources Studies, Tilburg University, Tilburg, Netherlands

**Keywords:** multi-teaming, multiple teams, multiple projects, multiple groups, bibliometric study

## Abstract

Multi-teaming is a concept studied across a variety of disciplines. While using a bibliometric approach on 255 research papers extracted from Web of Science, we aimed to depict the architecture of the multi-teaming concept across academic disciplines and time. Results of citation, co-citation and bibliographic coupling analyses identified four major fields looking at the concept of multi-teaming. The fields emerged over time from fragmentation to integration and acknowledging similarities. We identify gaps and propose (multi)-disciplinary research ideas that can benefit the field of multi-teaming.

## Introduction

The simultaneous grouping of agents into multiple teams, groups, projects, work settings and social entities is a ubiquitous phenomenon of our society (Raihani, 2021). Oftentimes we are simultaneously involved in multiple teams in work settings. In order to optimize the use of their human resources, modern organizations run multiple projects in parallel and deploy their employees to multiple teams in which they have to perform simultaneously. As such, multi-teaming raises important organizational (performance and well-being implications) and organizing (optimal structuring and scheduling of tasks) challenges. Therefore, in the past decades the concept of multi-teaming has been studied from various perspectives and disciplines.

In the Management and Applied Psychology fields multi-teaming has only recently started to capture the attention of researchers (e.g., the inclusion of single teams in research was considered to be the norm) (Mortensen et al., 2007; Mathieu et al., 2008). In these fields, multi-teaming was labeled as multiple-team membership and has been defined as “knowledge workers (…) being members of more than one project team at a time (O'Leary et al., 2011, p. 461), or "an employee's number of simultaneous and active team memberships, as reflected in the number of teams to which he or she allocates working time during a certain period” (van de Brake et al., 2020, p. 1505). The paper of O'Leary et al. (2011) was among the first to acknowledge multi-teaming as an important field of research that also has a strong practical, managerial relevance. It is estimated that 65 to 95 percent of knowledge workers belong to more than one team at a time (O'Leary et al., 2011) and 95% of middle and senior managers participate in more than one team at a time (Martin and Bal, 2007). The research focus in Management and Applied Psychology is to understand the impact of multiple team membership (as the dominant label) on individual, team, and organizational outcomes, with the ultimate goal of improving performance, well-being, and learning at different levels of analysis (O'Leary et al., 2011; Pluut et al., 2014; van de Brake et al., 2019, 2020).

In the Project Management sub-field (that is also part of the management field), multi-teaming is also extensively studied as a very common organizational phenomenon. Payne (1995) estimates that 90% of all projects are carried out in a multi-project context. The labels more often used are multi-project organization or multi-project teams. A multi-project organization has been defined as “an organizational unit that executes a substantial share of its operations as projects” (Engwall and Jerbrant, 2003, p. 403). This also implies that several projects are executed simultaneously (Gustavsson, 2016; Delisle, 2020) with the direct implication that employees are simultaneously allocated to multiple projects. Scholars within this field also put forward the idea that oftentimes project teams have to draw from the same pool of (limited) resources such as skills or engineering and design resources (Yaghootkar and Gil, 2012). The paper of Aritua et al. (2009) was among the first to acknowledge that the project management field in terms of guidance, tools and techniques was designed to support single project settings, while the majority of projects run in parallel and are actually organized in a simultaneous manner, asking employees to divide their time and resources across various tasks derived from these projects.

At the same time the concept of multi-teaming has also captured the attention of Operation Management and optimization and scheduling scholars. The scholars within this field take a prescriptive view on the concept of multi-teaming addressing the planning and organization challenges that emerge from multiple projects that run simultaneously in an (oftentimes) dynamic and complex environment (Song et al., 2018). The development of algorithms, heuristics and computational solutions addressing scheduling problems in multi-team settings are some of the dominant topics in this field (Kurtulus and Davis, 1982; Kurtulus and Narula, 1985; Lova et al., 2000; Lova and Tormos, 2001). As summarized above, multi-teaming is a concept studied across a variety of disciplines and sub-fields. One of the common elements of the definitions presented above is the idea of simultaneous allocation of individuals across multiple teams/projects. Hence, for the current study we propose the following encompassing definition for the phenomenon of multi-teaming: the simultaneous grouping of individuals into multiple teams, groups, projects, working and social entities.

Reviews addressing the concept of multi-teaming conducted so far looked at and summarized the literature within various fields. For example, the review of Margolis (2020) looked at the state of art of multiple team membership concept in the Management and Applied Psychology field. A recent special issue in International Journal of Project Management (Martinsuo and Geraldi, 2020) primarily focused on the studies published within the project management sub-field (e.g., actors, practices, and strategy connections in multi-project management).

In order to understand the phenomena of multi-teaming in a more comprehensive and holistic way we intend to look more in-depth at how it is represented and researched across various disciplines and fields/sub-fields, what are the major theories and conceptual frameworks, primary research questions and problem addressed and also if and the extent to which these fields are communicating with each other (Vogel et al., 2021). The current paper has thus three major goals. First, we aim to depict the architecture of the multi-teaming phenomena across various research fields. As such we intend to provide a tentative answer to the following questions: Which are the most important multi-teaming topics addressed in different research fields? Which are the most influential documents and journals in a particular research field? What is the intellectual structure of the field? Second, our paper intends to examine to what extent different multi-teaming fields and approaches are connected with each other, historically in the past and present. Third, we aim to identify gaps in the multi-teaming field and propose novel (multi-disciplinary) ideas for further investigation. The scope of our bibliometric study includes the field of multi-teaming, that is research that directly looks at instances in which employees are simultaneously part of more than one team or group.

The method used in this research is the bibliometric method (Zupic and Čater, 2015). This method is a science mapping technique that allows us to summarize large quantities of data and to present the intellectual structure and emerging trends of a research field (Donthu et al., 2021). When the scope of the review is broad, bibliometric analysis is a more suitable method as opposed to meta-analyses or systematic literature reviews as it is able to capture a broader spectrum of research articles compared to other methods (Donthu et al., 2021). By combining classification and visualization techniques, the bibliographic method allows us to depict the intellectual structure of a field and to show how disciplines, fields, specialties, or journals are related to each other (Zupic and Čater, 2015). In the current paper we are using three different techniques for science mapping, while looking both at scientific documents and journals as levels of analysis: citation analysis, co-citation analysis and bibliographic coupling. These techniques have been extensively described by Donthu et al. (2021) as well as by Zupic and Čater (2015). Such triangulation of various bibliometric methods provides a more comprehensive overview of complex multidisciplinary approaches that addressed multi-teaming, and each approach tackles different but complementary research questions, such in our case the past, present and future snapshots of multi-teaming (Premru et al., 2022).

## Sample/selection of papers

In line with previous recommendations regarding good practices in bibliometric methods (Chabowski et al., 2013; Zupic and Čater, 2015; Batistič and van der Laken, 2019) we approached a panel of 34 experts in the field and asked them for 4–5 representative keywords that best define the multi-teaming concept. With this step we aimed to increase the validity of our research. Of the 34 scholars approached 12 replied and their answers were further used in this research. The experts were selected from the three fields (e.g., 4 experts from Organization Behavior and Management, 5 experts from Project Management and 3 from Engineering, Operation Research) as our goal was to have a good representation of keywords across fields. In order to select the experts, we looked at relevant publications in the field and their academic impact (i.e., citations). The keywords that emerged and were subsequently used as part of the search strategy implemented in the Web of Science platform were: *multiple-team membership, multi-teams, concurrent teams, concurrent groups, multiple-team projects, multi-project, multi-project teams, multi project groups, concurrent project teams* and *concurrent project groups*. We searched for these keywords in the title field of Web of Science as this would elicit the most representative list of documents. We included the following types of documents that were listed in the Web of Science: research articles, books, book reviews, early access, editorial materials and review articles. We opted to exclude conference proceedings because these cannot be considered certified knowledge (Ramos-Rodríguez and Ruíz-Navarro, 2004). The initial search resulted in 332 hits. We read the abstracts of these documents and refined the list to 268 papers that refer to multi-teaming. We excluded for example literature on multi-team systems which refers to a different concept and other few papers that included the keywords in their title but did not refer to the multi-teaming concept within the scope of the manuscript. The final list included 255 documents, after excluding conference proceedings and one paper that did not have records regarding the authors. This sample was used for all three analytic sets reported below.

## Study 1. Citation analysis

Citation analysis looks at how primary papers in the database (the original papers that emerged from the search) relate to each other, thus it explores the flow of communication between documents in a network and tries to understand the influence of a document in the network in terms of citations they receive from other documents (Pieters and Baumgartner, 2002). One central concept in this analysis is cohesion. The more reciprocal relations and exchanges between the identified papers the higher the symmetry which indicates more cohesiveness in the field. The underlying assumption is that documents citing each other must be similar in terms of content or methodologies (Pieters and Baumgartner, 2002). For the citation analysis we used the VOSviewer (version 1.6.18) software tool (van Eck and Waltman, 2019).

**Figure 1:**
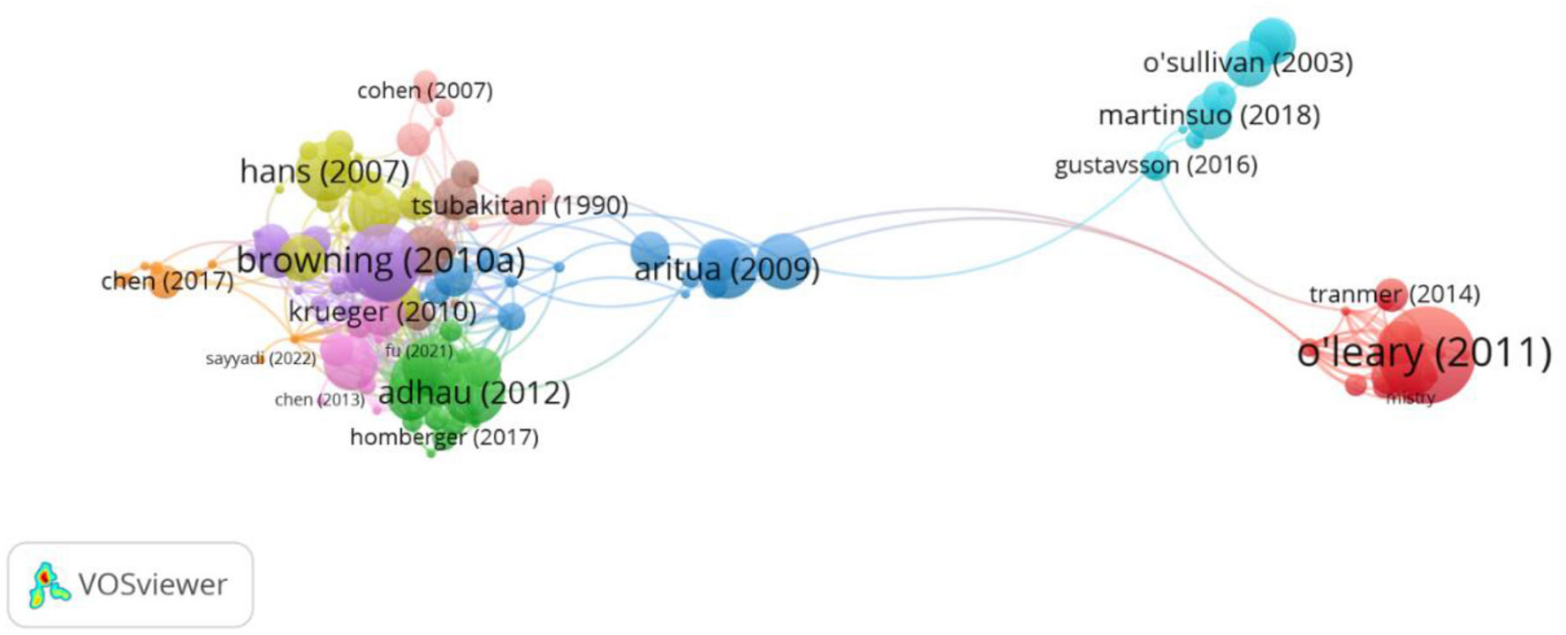
Citation analysis based on 134 documents.

In the first analytic set we performed a citation analysis based on papers as the level of analysis.^1^ We included all documents from our dataset irrespective of the number of citations of the documents. Out of 255 papers, 134 were connected and were included in this analysis. The analysis gives an overview of the field of the most influential papers. The VOS citation analysis yielded 10 different clusters (Figure 1). We focus on describing the first three more prominent clusters followed by an overall description of the network and relations among clusters. The first cluster (red) includes 21 papers, the majority of them in the field of Management, Applied psychology and Business. The papers were published in journals such as Journal of Applied Psychology, Academy of Management Review, Small Group Research, Journal of Organizational Behavior or Personnel Psychology. The paper of O'Leary et al. (2011) in Academy of Management Review appears to be the most influential in this sub-field with 198 citations.

The second cluster (green) includes 18 items, the majority of them in the field of Operations Research and Management Science, Industrial Engineering and Computer Science and Artificial Intelligence. The papers are published in journals such as International Journal of Production Economics, Annals of Operations Research, European Journal of Operational Research, Computers and Industrial Engineering. The papers approach the topic of scheduling in the context of multiple-teams/projects. The most influential paper here is the one of Adhau et al. (2012) published in Engineering Applications of Artificial Intelligence with 87 citations.

The third cluster (dark blue) includes 17 items, in the field of Management, Engineering and Economics. The papers are published in journals such as International Journal of Project Management, Journal of the Operational Research Society or Advances in Engineering Software. The papers deal mainly with the topic of multi-project management, with the most influential paper being the one of Aritua et al. (2009) in International Journal of Project Management with 78 citations.

When analyzing the network in a holistic manner we notice that on the left side we have a variety of clusters that connect well and are in the proximity of the green cluster. Here we see a variety of disciplines revolving around Operations Research and Management Science, Industrial Engineering and Computer Science. We identify topics such as multi-project planning (e.g., Hans et al., 2007; the most prominent paper in cluster 4) and multi-project scheduling mainly (e.g., Tsubakitani and Deckro, 1990; in cluster 9; Kumanan et al., 2006; in cluster 8; Krüger and Scholl, 2009; in cluster 7; Browning and Yassine, 2010; in cluster 5; Chen et al., 2017; in cluster 6).

In the middle of the network, we see that the project management cluster is well connected with Operations Research and Industrial Engineering fields (left side) as well as the Management and Applied Psychology cluster (the right side). Interesting to notice is that there is another project management cluster at the top right side, cluster 10 with 13 items (light blue). This cluster, together with the management one, is more marginal, having rather limited connections with the Operations Research and Industrial Engineering clusters.

**Figure 2:**
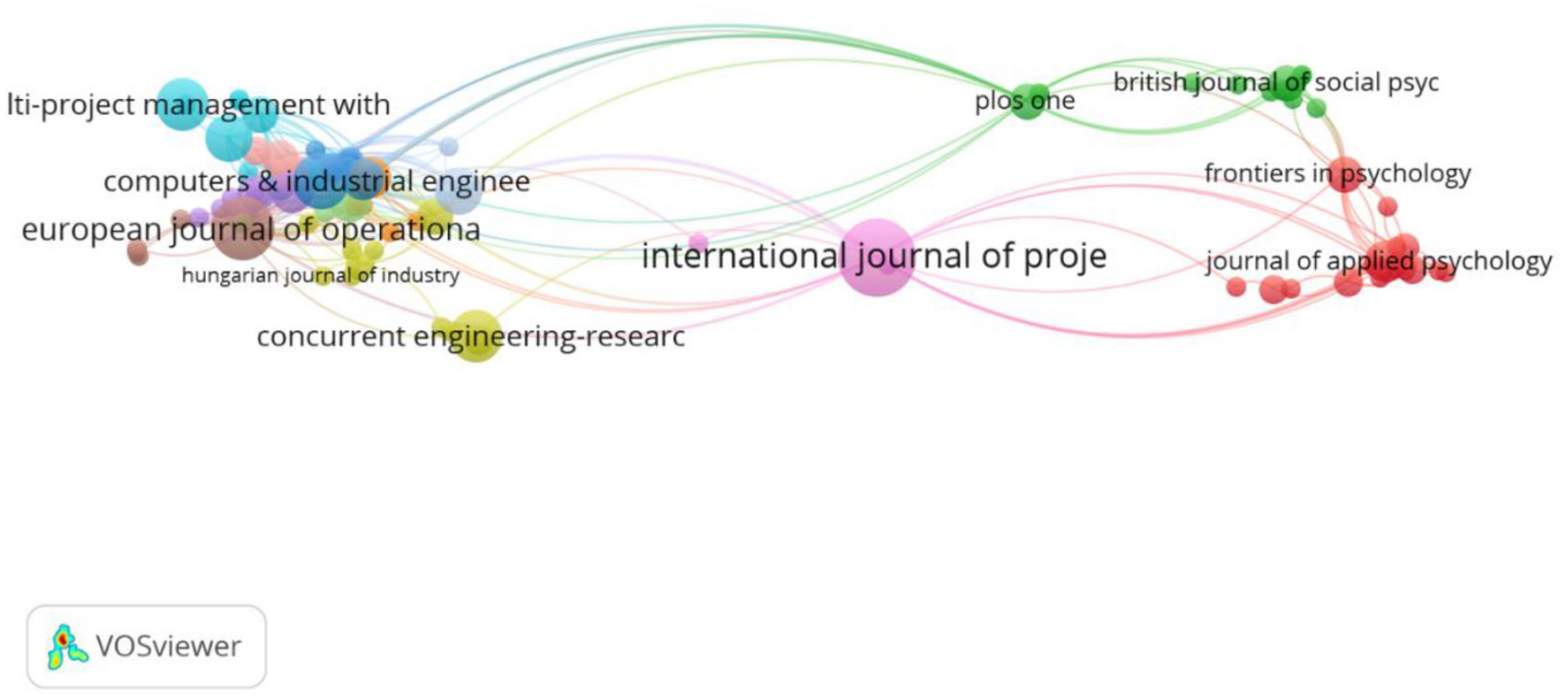
Citation analysis based on 98 journals.

Next, we performed a citation analysis based on source (journal), with the criteria of a minimum one paper per source. In this analysis, papers are grouped per journal, and the journal becomes the level of analysis. Ninety-eight connected sources emerged and were included in the analysis (see Figure 2). The first observation is that the International Journal of Project Management is the most central node in the citation network, with 13 papers published and 345 citations. Centrality also comes from the fact that it appears to connect well with the other subfields: Management and Applied Psychology on the right side and also Operations Research and Industrial Engineering on the left side. It is followed by European Journal of Operational Research with 9 papers and 279 citations and Computers and Industrial Engineering with 7 papers and 66 citations. 12 clusters emerged here. The first cluster (red, 19 journals) represents the Management and Applied Psychology field. Journals such as Academy of Management Review, Journal of Applied Psychology, Journal of Organization Behavior, as well as Frontiers in Psychology are represented here. Among others, the common studied themes in this cluster cover multi-teaming in relation to productivity (e.g., O'Leary et al., 2011), performance (e.g., van de Brake et al., 2018; Crawford et al., 2019; Rapp and Mathieu, 2019), (social) networks and networking (e.g., Tranmer et al., 2014; Mo and Wellman, 2016; van de Brake et al., 2020), collaboration (e.g., O'Sullivan, 2003; Matthews et al., 2012), coordination (Dietrich et al., 2013), and well-being (Pluut et al., 2014; Finuf et al., 2022).

The second cluster (green, 15 items) represents the Social Psychology, Gerontology, Psychiatry and Multidisciplinary Science fields. Journals included here are for example, British Journal of Social Psychology, Social Psychological and Personality Science, Aging and Mental Health, PLoS ONE. This stream of research, which we did not anticipate to emerge, looks at the phenomena of multi-teaming from a social psychological and sociological perspective. It reflects the extent to which a person is part of multiple social groups (e.g., community groups, sports groups) and not necessarily organizational groups like the other fields. The main topics encompassed here are the effects of being part of multiple social groups on individuals' (mental) health and well-being (e.g., Bule and Frings, 2015; Kinsella et al., 2020; Gallagher et al., 2021; Rees et al., 2022) or cognitive processing and performance (e.g., Woods et al., 2018; Beadleston et al., 2019). Furthermore, it also covers multiple group membership in relation to other social psychology themes, such as self-esteem (Jetten et al., 2015), aging (Ysseldyk et al., 2013), divorce (Lampraki et al., 2019), resilience in violence and abuse (Haslam et al., 2022), and protest (Besta et al., 2019). This cluster did not emerge in the first citation analysis, very likely due to the fact that the papers included in this cluster do not connect well with the other papers from the other clusters and hence were excluded from the sample used for the network.

The remaining clusters (represented on the left side of the network) are grouped around the Operations Research and Industrial Engineering field. Interesting to observe in this network is that International Journal of Project Management does not group to a particular field, although it is the most prominent journal, in which most of the multi-teaming papers have been published. At the same time, it is also a central actor, connecting well with the other sub-fields, which is evident also from the themes covered in the papers. These touch upon planning and scheduling (Caniëls and Bakens, 2012; Yaghootkar and Gil, 2012; Yang and Fu, 2014), information systems (Li et al., 2015), coordination (Hedborg et al., 2020), control (Laine et al., 2020), as well as learning (Chan et al., 2021), management (Martinsuo and Geraldi, 2020) and work overload (Gustavsson, 2016; Delisle, 2020).

## Study 2. Co-citation analysis

Co-citation analysis is the second analytic set and it refers to the frequency with which two secondary documents are co-cited by primary documents. The VOSviewer software creates a secondary dataset that includes all the documents that are co-cited together in the primary documents. The visualization is created based on this secondary dataset. The principle is that the more two documents are co-cited together, the more likely it is that they are semantically related and the more important they are in the field (Small, 1973; Zupic and Čater, 2015). This type of analysis reveals the underlying intellectual structure, theoretical foundations or the roots of a particular field (Zupic and Čater, 2015; Donthu et al., 2021).

**Figure 3:**
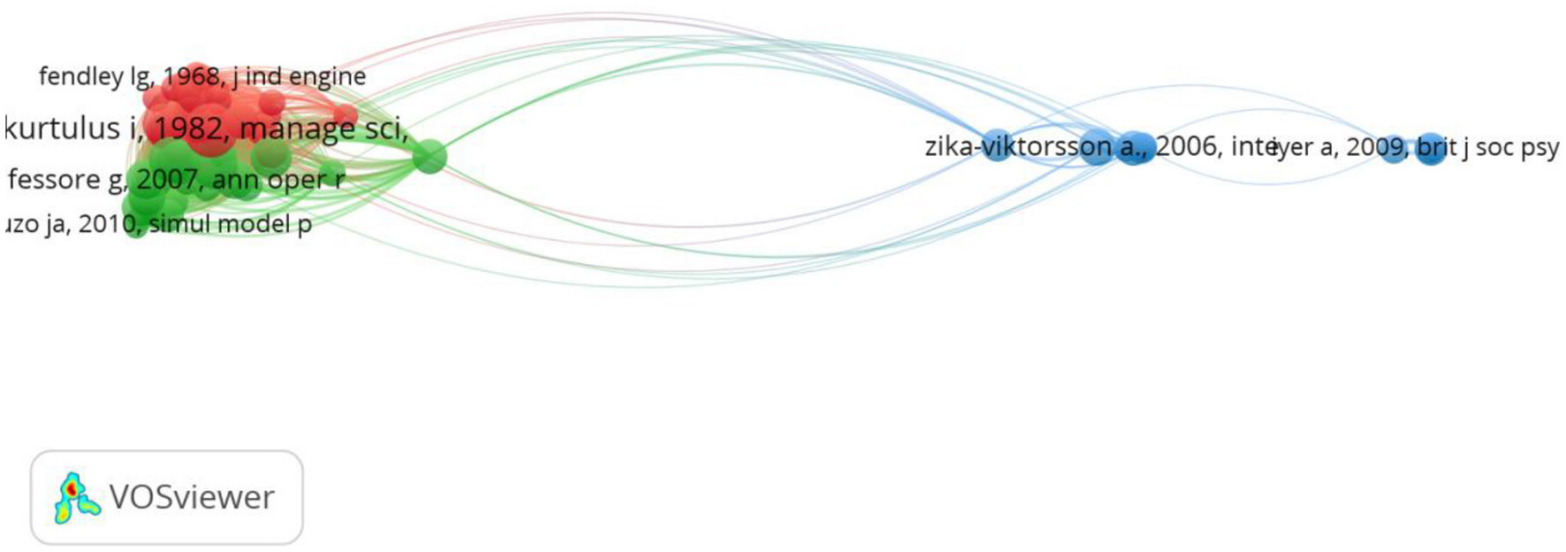
Co-citation analysis based on 59 papers.

First, we performed a co-citation analysis based on cited references, we limited the number of citations of a cited reference to 10, to facilitate the visualization.^2^ The sample resulted in 59 papers, with a total link strength (the frequency with which two secondary documents are co-cited by primary documents) varying from 472 to 25. Three clusters emerged (Figure 3). Clusters 1 (red) and 2 (green) on the left side of the graph cover mostly topics related to heuristics, meant to facilitate multi-project scheduling. They belong to the Operations Research and Industrial Engineering field. Although depicted as two separate clusters, their position in the network indicates that they are very closely related, possibly due to their topic similarity. On the right side, the third cluster (blue) represents the Management and Applied Psychology field. The main topics here relate to forms of multi-teaming (e.g., variety, number of teams) and their effects on organizational outcomes such as performance or stress. Another discussion in the field is whether multiple team membership represents a demand or a resource for the employees. A detailed description of the most prominent papers in each cluster can be found in Table 1.

**Table 1:** Top 5 most important documents for each cluster in the co-citation analysis.

**Cluster, color, field, *N* of items**	**Author(s), year, journal**	**Document description**	**Weight**
Cluster 1, red, Operations Research and Industrial Engineering (26 items)	(Kurtulus and Davis, 1982) Management Science	Application of heuristic solution procedures to multi-project scheduling.	417
	(Pritsker et al., 1969) Management Science	A zero-one (0-1) linear programming formulation of multiproject and job-shop scheduling problems is presented. This formulation is designed to accommodate a wide range of real-world situations (e.g., multiple resource constraints, due dates, job splitting, resource, substitutability, and concurrency and nonconcurrence of job performance requirements). It provides a solution for multi-project scheduling with limited resources.	293
	(Lova et al., 2000) European Journal of Operational Research	A multicriteria heuristic method to improve resource allocation in multiproject scheduling is developed. The multicriteria heuristic algorithm consists of several algorithms based on the improvement of multiproject feasible schedules. Through an extensive computational study, it is shown that this method improves the feasible multiproject schedule obtained from heuristic methods.	214
	(Kurtulus and Narula, 1985) IIE Transactions	Applications of heuristic solution procedures to the resource-constrained, multi-project scheduling problem is examined. Depending on the characteristics of the multi-project scheduling problem, various scheduling rules can be applied.	210
	(Brucker et al., 1999) European Journal of Operational Research	A classification scheme for the resource-constrained project scheduling is provided, a unifying notation is proposed, and the exact heuristic algorithm used in the literature is reviewed.	207
Cluster 2, green, Operations Research and Industrial Engineering field (21 items)	(Lova and Tormos, 2001) Annals of Operations Research	A new heuristic – based on priority rules with a two-phase approach – that outperforms the classical ones, is proposed. This new heuristic helps minimize mean project delays in a multi-project setting.	403
	(Gonçalves et al., 2008) European Journal of Operational Research	A genetic algorithm for the resource constrained multi-project scheduling problem is presented. Computational results prove the effectiveness of the algorithm.	386
	(Confessore et al., 2007) Annals of Operations Research	A market-based multi-agent system model for decentralized multi-project scheduling is proposed.	317
	(Homberger, 2007) International Transactions in Operational Research	A restart evolution strategy for the resource-constrained project scheduling problem is presented. This is also integrated into a multi-agent system to be able to solve the decentralized resource-constrained multi-project scheduling problem.	281
	(Kolisch, 1996) European Journal of Operational Research	A computational study in which the parallel and serial scheduling method is reconsidered for the classical resource-constrained project scheduling problem.	277
Cluster 3, blue, Management and Applied psychology (11 items)	(Zika-Viktorsson et al., 2006) International Journal of Project Management	An empirical study conducted in Sweden on 392 project workers. Results show that almost a third of project workers perceive project overload; which is due to diminished opportunities for recuperation, inappropriate routines, scarce time resources and an extensive number of simultaneous projects. Moreover, study finds associations between high project overload and psychological stress, diminished competence development, as well as changes of time schedules.	68
	(O'Leary et al., 2011) Academy of Management Review	A model in which the effects of the number and variety of multiple teams on productivity and learning at two different levels of analysis: individual and team level, is proposed.	67
	(Cummings and Haas, 2012) Journal of Organization Behavior	The antecedents and consequences of member time allocation in a multi-level study of 2055 members of 285 teams in a large global corporation is investigated. Results at the individual level show that time allocation is influenced by members' levels of experience, rank, education, and leader role on the team. Furthermore, the effects of member time allocation on team performance depends on geographic dispersion.	58
	(Pluut et al., 2014) Group Dynamics: Theory, Research, and Practice	Drawing on the Job Demands–Resources framework and role theory the study looks at whether fragmentation of time across teams is a job demand or a job resource for employees. The findings show that multiple team membership increases demands associated with teamwork but not taskwork, while at the same time reducing social support from team members.	58
	(Bertolotti et al., 2015) Research Policy	An inverted U-shaped relationship between multiple team membership and team performance was found, such that teams whose members are engaged simultaneously in few or many teams experience lower performance. Receiving advice from external sources and the use of instant messaging moderates this relationship.	54

The weight column indicates the total strength of the co-citations links of a given document with other documents. The higher the weight, the more important a document is to the network.

**Figure 4:**
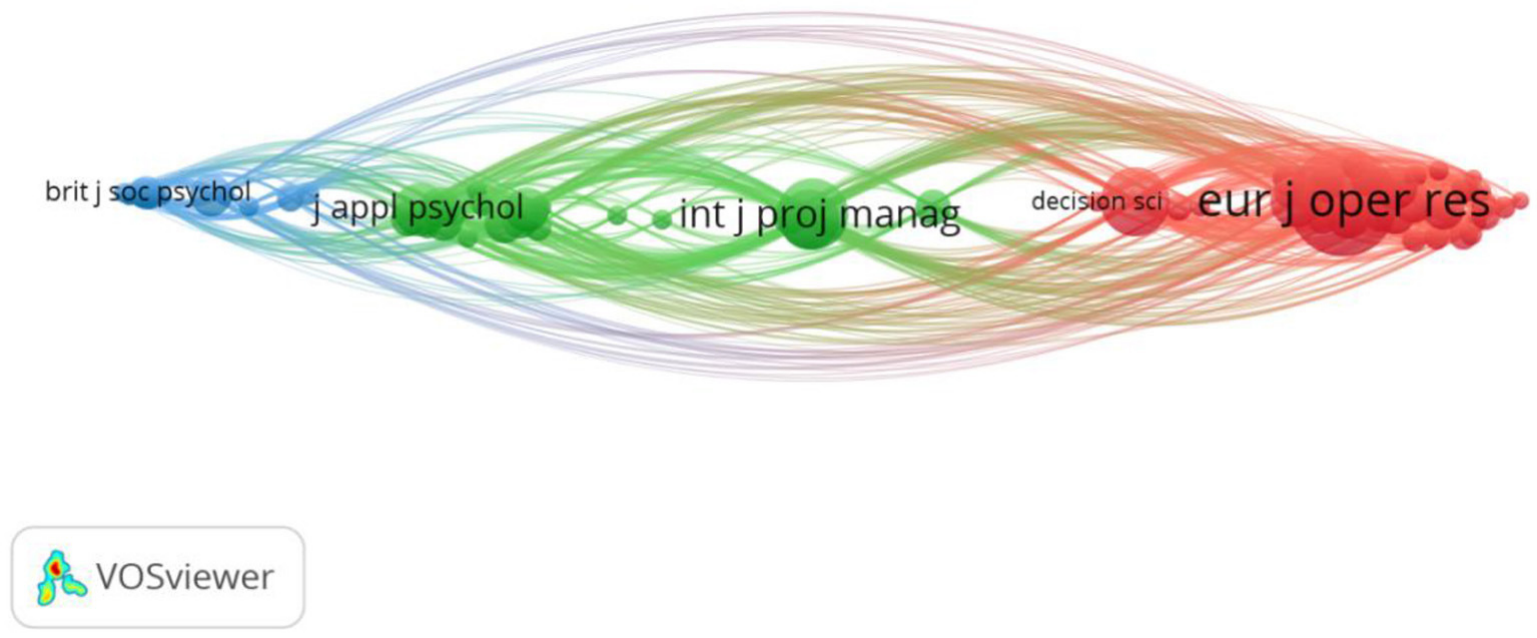
Co-citation analysis based on source (67 sources).

Next, we performed a co-citation analysis while using the source (journal) as a level of analysis. As a criteria we used a minimum of 20 citations per source to have a better visualization of the field. A number of 67 sources were included. The results show that 3 main clusters emerged (see Figure 4). The first cluster (red) includes 32 items and reflects the Operations Research and Industrial Engineering field, aligned with previous findings. In the middle, the second cluster (green) represents the Management and Applied Psychology field with 20 sources. Finally, cluster 3 (blue) represents the Social Psychology, Psychiatry field. Interesting to observe that International Journal of Project Management plays a very central role connecting well with both Cluster 1 and Cluster 3.

## Study 3. Bibliographic coupling

Bibliographic coupling is the third set of analyses and it focuses on the primary documents and the extent to which their bibliographies overlap, thus it captures the extent to which pairs of papers share common references. The larger the overlap between the bibliographies of two papers, the larger the coupling strength is between them. This type of analysis is focused on identifying current trends given that primary documents are more recent than cited papers. Hence, this type of analysis is indicative of current trends in the field and future directions of the field (Vogel et al., 2021).

First, we performed a bibliographic coupling analysis based on the papers. As a threshold, we used a number of 5 citations per document. This inclusion criterion limited the number of primary documents to a manageable size, leading to a clearer visualization (Zupic and Čater, 2015). This resulted in a network composed of 130 items. The document with the highest coupling strength is the book of Walter (2015), Multi-Project Management with a Multi-Skilled Workforce, with a total link strength of 4,069. The book covers the topic of optimization in managing multiple project teams. This is followed by the paper of Seddon et al. (2010), in MIS Quarterly (total link strength of 3,029), where they develop and test a long-term, multi-project model of factors affecting organizational benefits from enterprise systems. The third paper is the one of Homberger (2012) (total link strength 1,873), where the author looks at a coordination mechanism for agent-based multi-project scheduling.

**Figure 5:**
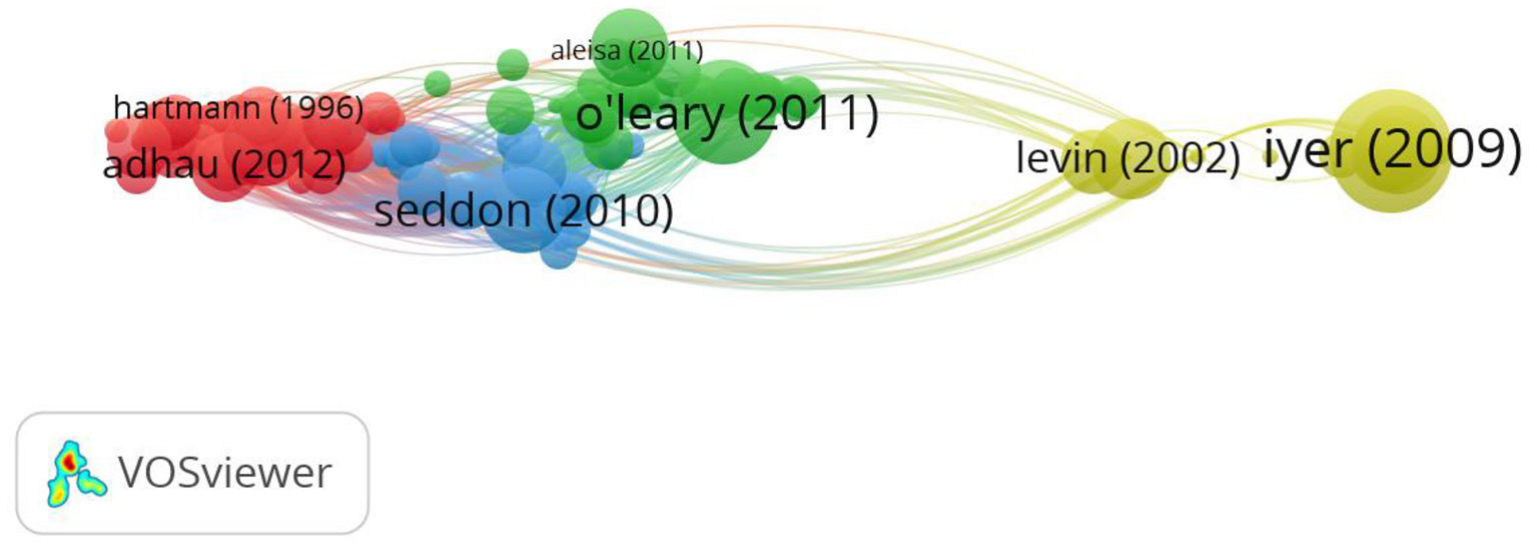
Bibliographic coupling analysis based on documents.

Four clusters emerged here (Figure 5). The first cluster (red) includes the Operations Research and Industrial Engineering field. The second cluster (green) is a mix of Computing and Industrial Engineering, Applied Psychology, (Project)/Management. The third cluster (blue), also the one that is the most strongly connected with the other ones, likewise includes a mix of Operations Research, Project Management and Industrial Engineering. The fourth cluster (yellow) represents the Social Psychology field. A description of the 5 most connected papers within each cluster can be found in Table 2. The main observation here is that in comparison to the citation and co-citation analyses, the main fields identified (Operation Management, Computing and Industrial Engineering, Management and Applied Psychology) come closer together, mingle within clusters, and are overall closely represented within the network.

**Table 2:** Top 5 most important documents for each cluster in the bibliographic coupling analysis.

**Cluster, color, field, *N* of items**	**Author(s), year and journal**	**Document description**	**Weight**
Cluster 1, red, Operations Research and Industrial Engineering (53 items)	(Can and Ulusoy, 2014) Annals of Operations Research	A genetic algorithm approach for multi-project scheduling with two-stage decomposition is presented.	986
	(Krüger and Scholl, 2009) European Journal of Operational Research	A heuristic solution framework for the resource constrained (multi-)project scheduling problem with sequence-dependent transfer times is proposed.	687
	(Browning and Yassine, 2010) International Journal of Production Economics	A comprehensive analysis of 20 priority rules on resource allocation in order to curtail average project delay, is conducted. Contexts in resource-constrained multi-project scheduling in which these rules are effective and in which not, are identified.	683
	(Suresh et al., 2015) Asia-Pacific Journal of Operational Research	A new genetic algorithm approach to the multi-project scheduling problem with resource transfer times is developed and discussed in this paper.	469
	(Adhau et al., 2012) Engineering Applications of Artificial Intelligence	An auction-based negotiation approach to distributed multi-project scheduling is discussed.	362
Cluster 2, green, Computing and Industrial Engineering, Applied Psychology, (Project)/Management (41 items)	(Componation and Byrd, 2000) IEEE Transactions of Engineering Management	Cluster analysis is used to structure concurrent engineering teams.	310
	(Crawford et al., 2019) Journal of Applied Psychology	Empirical study in which multiple team membership is negatively associated with unit performance, and this negative relationship is further increased by task complexity.	253
	(O'Leary et al., 2011) Academy of Management Review	A model in which the effects of the number and variety of multiple teams on productivity and learning at two different levels of analysis: individual and team level, is proposed and discussed.	232
	(Caniëls and Bakens, 2012) International Journal of Project Management	The effects of Project Management Information Systems on decision making in a multi project environment are discussed.	221
	(Vuorinen and Martinsuo, 2018) International Journal of Project Management	The topic of program integration in multi-project change programs is approached.	183
Cluster 3, blue, Operations Research, Project Management and Industrial Engineering (18 items)	(Walter, 2015) Multi-Project Management with a Multi-Skilled Workforce	Three fundamental problems in multi-project management are considered in this book: the selection of projects, the composition of small project teams, and workload leveling. Optimization models and solution methods for these problems are advanced.	4,069
	(Seddon et al., 2010) MIS Quarterly	A long-term, multi-project model of factors affecting organizational benefits from enterprise systems is developed.	3,029
	(Homberger, 2012) OR Spectrum	A new generic negotiation-based mechanism to coordinate project planning software agents to share resources among projects is described.	1,873
	(Voß and Witt, 2007) International Journal of Production Economics	A mathematical model to address a real-world multi-mode multi-project scheduling problem is proposed.	1,589
	(Aritua et al., 2009) International Journal of Project Management	A complex adaptive perspective to multi-project environment with an application to construction industry is advanced.	751
Cluster 4, yellow, Social Psychology (18 items)	(Woods et al., 2018) Social Neuroscience	The relationship between rejection sensitivity and depressive symptoms is investigated and mediation by multiple group memberships was found. This relation was only evident among individuals possessing a particular type of gene configuration.	184
	(Levin et al., 2002) Psychological Science	The joint impact of gender and ethnicity as different social groups on expectations of general discrimination against oneself and one's group is examined. The results reveal that women of color do not differ from man of color when considering their expectation of general discrimination. This is due to the increased perception of ethnic, rather than gender discrimination.	154
	(Andriessen et al., 2012) Work and Occupations	Ethnic discrimination in the Dutch labor market is addressed and relation with job characteristics and multiple group membership is considered. Results indicate that ethnic minorities experience discrimination on the Dutch labor market and there is no differentiation between these minority groups. However, migrant men are exposed to more discrimination than migrant women.	153
	(Jetten et al., 2015) PLoS ONE	Belonging to multiple important group memberships is shown to predicts personal self-esteem in children, older adults and former residents of a homeless shelter. Multiple important group memberships also predict personal self-esteem over time.	93
	(Cruwys et al., 2016) British Journal of Social Psychology	Social Identity Mapping as a method for visually representing and assessing a person's subjective network of group memberships is introduced.	88

The weight column indicates the total strength of the co-citations links of a given document with other documents. The higher the weight, the more important a document is to the network.

**Figure 6:**
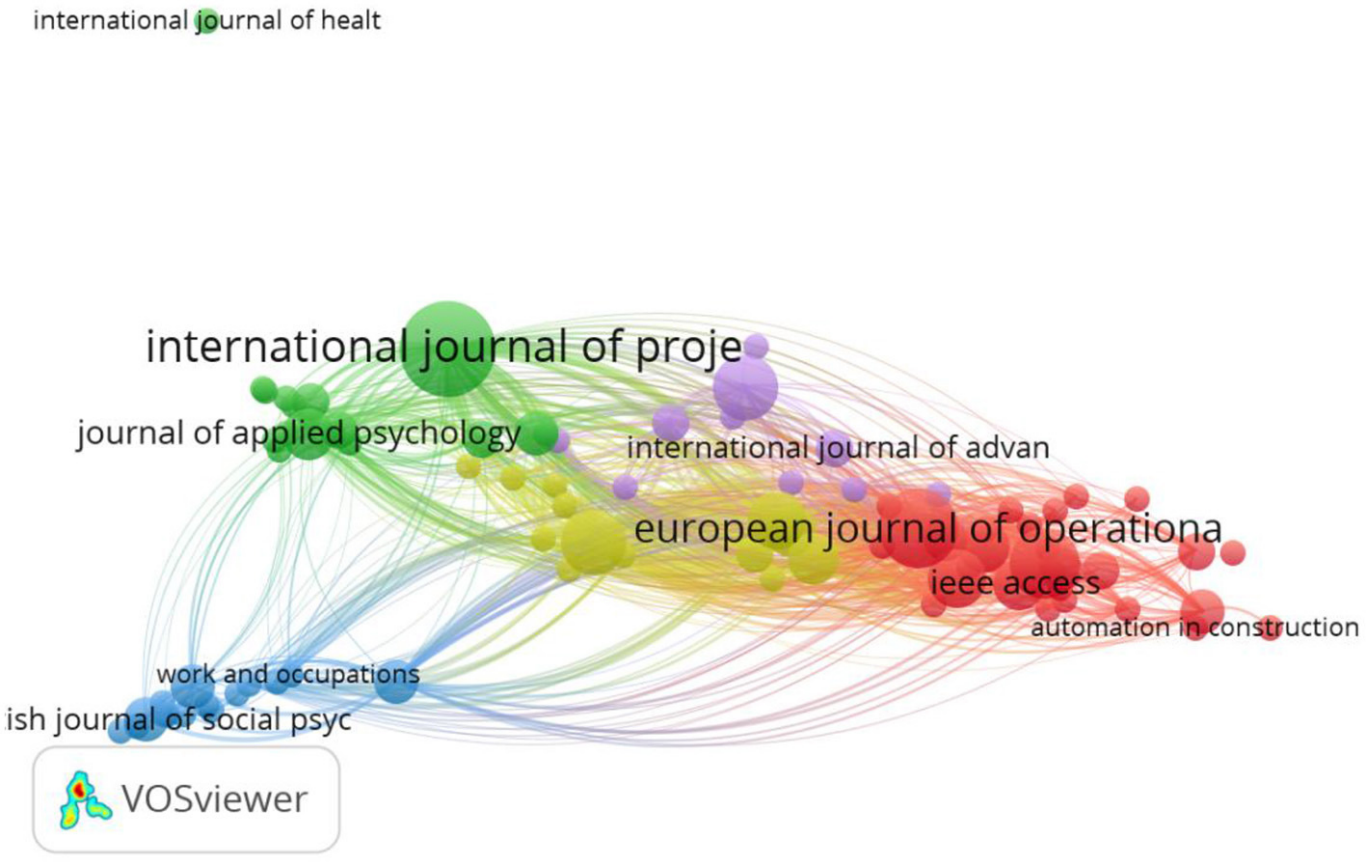
Bibliographic coupling analysis based on source.

The last analysis performed is a bibliographic coupling analysis based on source or journal. We used as a criteria a minimum of 5 citations per source. This resulted in 85 items organized in 5 clusters (Figure 6). The red cluster (29 items) represents mostly the Operation Management, Computing and Industrial Engineering fields. The green cluster (18 items) represents the Applied Psychology and Management fields. The blue cluster (15 items) represents the Social Psychology sub-field and the multi-group literature. The yellow (13 items) and the purple clusters (10 items) is a mix of Operation Management and Computing and Industrial Engineering.

What we observe, also in line with the document analysis, is that the main fields of Operation Management, Computing and Industrial Engineering, Management and Applied Psychology are closely connected to each other, while the emerging Social Psychology field has a more marginal position. Also, the International Journal of Project Management is still the most prominent, with 13 papers and 345 total citations followed by the European Journal of Operational Research with 9 papers and 279 citations.

## Theoretical contributions, limitations and directions for future research

The current paper aimed at investigating the ways in which multi-teaming phenomenon is conceptualized and studied across different research fields. In what follows we provide a detailed discussion of the analyses pertaining to the three main goals of the paper. We discuss first how the architecture of the multi-teaming phenomenon across disciplines looks like, how the different fields are connected with each other (in the past and present) and finally we discuss the research gaps and how the multi-teaming field can be advanced while proposing future multi-disciplinary directions for research. Collaboration across disciplines has the value of advancing a research field by offering new ways of looking at one phenomenon and by shedding light on particular observations where no adequate explanations exists (Shaw et al., 2018).

### The architecture of the multi-teaming phenomena across different research fields (Goal 1)

The citation analysis revealed that the concept of multi-teaming spans across three major fields and one sub-field. The first field is the one of Management, Applied Psychology and Business. Journals such as Journal of Applied Psychology, Academy of Management Review, Small Group Research, Journal of Organizational Behavior or Personnel Psychology are representative here. The key paper in this cluster is the one of O'Leary et al. (2011) in the Academy of Management Review, with 198 citations. This is an original theoretical model regarding how multiple-team membership is expected to influence learning and productivity at the individual and team level. The paper also discusses various ways in which multiple-team membership can be operationalized such as the number of teams and variety of teams. Given that it is one of the first theoretical models in this sub-field, it emerged as one of the most prominent paper in the cluster.

Papers in this cluster looked at the phenomenon of multi-teaming from multiple angles. One important question that drew researchers' attention was whether multi-teaming can be seen as a resource or as a demand in working settings. In this regard studies have relied on the Job Demands-Resources model (Bakker and Demerouti, 2007) to identify that fragmentation across multiple teams for example is a teamwork-related job demand and leads to role strain (Pluut et al., 2014). Also, being part of multiple teams is associated with job demands and this relation is moderated by polychronicity and support (Finuf et al., 2022). For employees with low organizational tenure, being part of multiple teams is negatively associated with perceived work challenge and positively associated with role ambiguity which in turn lead to diminished job performance and more absenteeism (van de Brake et al., 2020). Another angle is represented by the social network theories (Reagans and Zuckerman, 2001) and also the social capital perspective (Lin, 1999, 2001). These theories emphasize that being part of multiple teams exposes individuals to a greater number of team members and this provides access to a wide variety of resources (e.g., information) and ultimately fosters learning and productivity (O'Leary et al., 2011). In line with these theories it has been shown empirically that being part of multiple teams leads to increased job performance when the information-sharing network is large (van de Brake et al., 2020). Also being part of multiple (varied) teams leads to increased individual learning (Chan et al., 2021). Teams whose members belong to multiple inter-organizational teams are also more likely to increase their external learning (Chan et al., 2021).

Another stream makes use of the attention-based theories. These theories emphasize that an increasing number of demands compete for people's attention (Hansen and Haas, 2001; O'Leary et al., 2011). Being part of multiple teams fragments attention as individuals switch between team contexts and they need to invest additional time and effort to switch off and on from one context to another. Crawford et al. (2019) found that MTM is negatively associated with unit performance and this relation is exacerbated by task complexity.

Another line of research is connected with role theory, or the idea that individuals held situation-specific roles that invoke expectations for their behaviors (Kauppila, 2014). Participating in multiple roles (especially when these roles are different) might lead to role strain, due to the fact that individuals are confronted with different and sometimes conflicting role obligations (Goode, 1960). Empirical research identified that role separation (or the extent to which there are differences between an individual's role in a focal team and his or her roles in other teams) in a multi-team setting leads to role ambiguity which can further harm the performance of the team (van de Brake and Berger, 2021). Related, the role identity theory (Burke, 1991) suggests that work roles involve activities and expectations that contribute to an individual's identity (Stryker and Burke, 2000). Being part of multiple teams can generate difficulties in establishing a coherent social identity and this can further lead to identity-related strain (Mistry et al., 2022).

Identity conflict in multi-team setting (as the competition, conflict and contradiction among different identities a team member is part of) was connected to decreased innovative performance (Chen et al., 2021). Rapp and Mathieu (2019), point toward the importance of team identity and they show that when individuals are assigned to multiple teams they develop unique identities for each; furthermore, these teams identities work as mediators between various individual and team antecedents (e.g., role stressors, team cohesion) and individual level team performance.

The second field identified in our bibliometric analysis includes Operations Research and Management Science, Industrial Engineering, and Computer Science and Artificial Intelligence. Journals such as the International Journal of Production Economics, Annals of Operations Research, European Journal of Operational Research or Computers and Industrial Engineering are representative here. European Journal of Operational Research is the second most prominent journal in the network with 9 papers and 279 citations, followed by Computers and Industrial Engineering with 7 papers and 66 citations. The papers within this cluster deal almost exclusively with the topic of scheduling and optimization in the context of multiple-teams/projects. The most influential paper here is the one of Adhau et al. (2012) published in Engineering Applications of Artificial Intelligence with 87 citations. The study proposes a new multi-agent system approach that solves some of the issues associated with the allocation of resources across multiple competing projects. Research in this field looks normatively at the multi-project scheduling problem where resources are limited and explores ways in which it can be optimized. The study of Liu et al. (2021) developed a scheduling algorithm that takes into account resource disruptions, the study of Fu and Zhou (2021) developed a combined multi-agent system for distributed multi-scheduling problem that proved to be better than a central coordination mechanism. The study of Li et al. (2021) proposes a multi-agent based cooperative approach with a negotiation protocol that leads to an efficient global allocation of resources, and Liu and Xu (2020) develop a heuristic that considers the uncertain duration in scheduling distributed multi-projects. Other elements are also considered in this setting, such as asymmetric information and opportunistic behavior (Homberger, 2012). In terms of frameworks used most of the papers rely on auction-based mechanisms (Rothkopf et al., 1998), negotiation-based mechanisms (Homberger and Fink, 2017) and/or game theory approaches (Agnetis et al., 2015). In general, studies within this field tackle the issue of scheduling in multi-team settings while proposing optimized solutions that integrate various parameters modeled from the organization.

The third identified field is the one of project teams, spanning across Management, Engineering and Economics. The papers deal mainly with the topic of multi-project management, with the most influential paper being the one of Aritua et al. (2009) in International Journal of Project Management with 78 citations. The paper uses a complex adaptive systems perspective to discuss multi-project management while giving an example in the UK construction industry. Journal such as International Journal of Project Management, Journal of the Operational Research Society or Advances in Engineering Software are central here. Besides, International Journal of Project Management is the most central actor in the field, with 13 papers published and 345 citations. Since multi-project management is (theoretically) part of the management field, it can also be categorized as a sub-field of management. Interestingly, two clusters emerged here. The first one is connected with the topic of scheduling in multi-project environments, when resources are limited. The main logic here is that in order to remain competitive, firms have to constantly improve their current products and develop new ones; in order to do so, they make use of a multi-project environment where teams have to share engineering and design resources (Yaghootkar and Gil, 2012). Researchers are interested in identifying which methods, models, decision trees or managerial practices could benefit organizations using a multi-project setting. The paper of Yang and Fu (2014) for example proposes a new multi-project schedule method that can be used by project managers which is based on task priority, evidence reasoning and a critical chain approach. Yaghootkar and Gil (2012) identify that a schedule-driven project management policy is not beneficial for the overall organizational performance on a long run given that investing resources in some projects deprives the other remaining projects and staff switches across projects reduce productivity. Caniëls and Bakens (2012) look at the role of project management information systems and find that their information quality is positively related to the quality of decisions and satisfaction of the project managers. Some other papers look more in depth at models that tackle the time-cost trade off problem (Taheri Amiri et al., 2018), or the optimization of multi-project scheduling on the critical chain while also considering multiple objectives (Wang et al., 2014).

The second cluster revolves around the special issue of Martinsuo et al. (2020) on multi-project management. Studies here look at practices that contribute to work overload in a multi-project setting (Delisle, 2020), how teams can coordinate multi-team projects (Dietrich et al., 2013; Hedborg et al., 2020), how task interdependencies can be managed in multi-team projects (Hoegl and Weinkauf, 2005), or the role of formal and informal networks in balancing creativity and efficiency in multi-team projects (Kratzer et al., 2008). The main theories papers in the second cluster rely on come from management and organizational studies, such as structural contingency theory (Lawrence and Lorsch, 1967) or structuration theory (Giddens, 1984; Jarzabkowski, 2004). The fourth field identified is represented by Social Psychology, Gerontology, Psychiatry and Multidisciplinary Science. British Journal of Social Psychology, Social Psychological and Personality Science, Aging and Mental Health, PLoS ONE are representative journals here. This stream of research looks at the phenomena of multi-teaming from a social psychological perspective and it is a field that was not anticipated and emerged from our bibliometric analysis. The most frequently used keyword is ”multiple group membership”, which represents the extent to which an individual is part of multiple social groups (e.g., community groups; sports groups) (Gallagher et al., 2021). The Social Identity Theory (Tajfel and Turner, 1979; Tajfel, 1982) and also Self-Categorization Theory (Turner et al., 1987) that explain how membership in various social groups (family, community, religious, sporting) shape social identity or the way in which we develop our sense of self and we derive self-esteem from these memberships (Haslam et al., 2022). Multiple group membership benefits the health and well-being of individuals because they have access to multiple social identities that keep them connected to other people, provide structure and meaning to social relationships (Greenaway et al., 2016). For example, the study of Bule and Frings (2015) shows the positive role (for patient well-being) of maintaining group memberships post-operative/after surgery. In the context of acquired brain injury belonging to multiple groups lowered depression symptoms because exposure to multiple groups gave participants the chance to practice and develop self-regulatory skills (Kinsella et al., 2020). The study of Ferris et al. (2016) also showed that being part of multiple groups leads to an increase in communication about pain and this communication reduced brain activation in regions associated with pain. Being part of multiple groups is in this context, a resource that diminishes pain. The study by Berry et al. (2019) found that young people not in employment, education and training are more depressed and one of the mechanisms explaining this is their lack of multiple group memberships. Also being part of multiple groups reduced the feeling of loneliness among divorced people in the long run (Lampraki et al., 2019). In this field, being part of multiple groups is viewed as a social support system that benefits individuals, in terms of quality of life, health and well-being.

When it comes to the intellectual structure of the field, based on the co-citation analysis, two major streams emerged. The first stream deals with the problem of multi-project scheduling. Topics such as heuristic procedures (Kurtulus and Davis, 1982; Kurtulus and Narula, 1985; Lova et al., 2000; Lova and Tormos, 2001), algorithms (Gonçalves et al., 2008), multi-agent system models (Confessore et al., 2007; Homberger, 2007) in the context of resource constrained multi-project scheduling problem are some of the intellectual foundations of the multi-teaming field. The second stream that emerged relates to identifying antecedents and consequences of multi-teaming, more from an organizational behavior and management perspective. Topics such as project overload and its effects on stress (Zika-Viktorsson et al., 2006), effects of multi-teaming on productivity and learning (O'Leary et al., 2011), antecedents and consequences of time allocation across multiple teams (Cummings and Haas, 2012), the effects of multiple team membership on teamwork and taskwork (Pluut et al., 2014), and performance (Bertolotti et al., 2015) are fundamental here.

### Connections among multi-teaming fields and disciplines in the past and present (Goal 2)

The second goal of the paper was to look at the extent to which various disciplines identified in the bibliometric analyses communicate with each other, while looking at the past and present. While citation and co-citation analyses give an overview of the past (it takes time for a paper to get cited and hence older papers have higher chances to become influential), the bibliographic coupling method focuses on the present, and the current research front (Vogel et al., 2021).

When looking at Figure 1 from the citation analysis, we observe that the different fields in multi-teaming are differentiated. The Management and Applied Psychology fields have a marginal position in the right lower corner (red cluster) with few connections to the project management field mainly. The papers situated in the Management and Applied Psychology field are in general rich in terms of conceptualization and they also make in-depth use of a variety of theories, which is also one of the main contributions of this field. This explains its connections with the second cluster of the project management field which also has a focus on management and organizational theories. The project management is split into two clusters (the dark blue in the middle and the light blue on the right upper corner). This split is explained by the fact that this field is able to accommodate a variety of angles toward the multi-teaming phenomenon, and this is also one of the contributions it has to the field. On the one hand it includes topics related to methods, models, decision trees or managerial practices used in multi-teaming and hence it's connections to the Operations Research field; on the other hand it includes organizational-related topics (e.g., workload) and hence its connections to the Management field.

The clusters around the Operations Research and Management Science field, on the left side of Figure 1, are close to each other and also well connected. Most of the papers in this field look at specific algorithms and heuristics meant to optimize scheduling in a multi-team setting; this topic specifically explains why the papers and clusters are so cohesive. The main contribution of this field is that it addresses a very practical problem (the scheduling one), oftentimes with very specific recommendations for management. In this respect it also resembles the first project management cluster and differentiates itself from the Management and Applied Psychology field that puts a greater emphasizes on theories. Important to notice is that the project management cluster in the middle is the only one connecting well with all the other disciplines, hence its central position. Interestingly, when we look at the intellectual structure of the field (Figure 3) we observe that Operations Research and Management Science field clusters at the left side and the Management and Applied Psychology fields cluster at the right side. The project management sub-field is distributed across these three clusters and hence its central position in Figure 4, which is a co-citation analysis based on journal. Based on these results, we can claim that from a historical point of view, project management field is the bridging link between the Operations Research and Management Science field, and The Management and Applied Psychology fields.

Finally, when looking at the bibliometric coupling analysis (Figures 5, 6), focusing on the present time, we observe that the distance between the sub-fields is reduced. Within each cluster, we have a mix of disciplines, such as Operation Management, Computing and Industrial Engineering, Management and Applied Psychology. This is an indicator that in time scholars have started to acknowledge the similarities across the different fields. The only field that remains more (although not completely) disconnected from the rest is the one of Social Psychology (yellow cluster in Figure 5 and blue cluster in Figure 6). This field is strongly anchored in the social identity theory, and hence its connections with the Management field that also makes use of this theory. The main contribution of this field is to show that multi-teaming can be successfully used as a social intervention that improves health and well-being. The concept of multi-teaming is not necessarily extensively theorized or explained, however, the field introduces detailed ways in which it can be measured (e.g., the social identity mapping measure of Cruwys et al., 2016), and hence an additional contribution.

### Gaps and directions for future research (Goal 3)

One notable strength of the bibliometric approach is that is has the potential to uncover existing gaps in a particular research field and guide directions for future research (Bunjak et al., 2022). While focusing our analysis on both documents and journals we obtained a comprehensive picture of how the concept of multi-teaming is represented across different fields, given that journals are categorized as belonging to different disciplines. Based on this overview we identify potential gaps and make recommendations for future research. The main focus of our recommendations is to increase communication and cross-fertilization across different research fields. Integration across research fields has the potential to advance a particular field by bringing novel insights and shedding light on phenomena that are difficult to explain otherwise (Shaw et al., 2018).

When looking at the specific connections among the fields one observation that emerges is that the Social Psychology, Gerontology, Psychiatry and Multidisciplinary Science field remains disconnected (more so in the past but also in the present) from the other emerged fields. The reasoning may lie in the fact that in contrast to Management, Project Management, Applied Psychology, Operations Research and Management Science, Industrial Engineering, and Computer Science and Artificial Intelligence fields, which predominantly focus on work related topics, this field approaches the topic of multi-teaming from a social perspective, that is multiple groups that people have and engage in outside of their working environment. The same concept of multi-teaming has been studied in two different settings (work setting and social/personal setting) without sufficient acknowledgment of their similarities or possible connections. Given this gap we propose a first research direction that integrates the Social Psychology stream with the Management/Applied Psychology stream (Table 3). This stream can look at important research questions such as the effects of multi-teaming (both organizational and social/personal sphere) on individual well-being, organizational and team outcomes. It could also establish if multi-teaming is a phenomenon generalizable across settings (e.g., individual characteristics that predict multi-teaming in organizational settings also predict multi-teaming in personal/social settings and the other way around). Simultaneously looking at multi-teaming across settings brings a better understanding of the effects of multi-teaming on individual and organizational outcomes.

**Table 3:** Examples of (multi-disciplinary) directions for future research.

**Multi-disciplinary research/Communication between Fields**	**Examples of research ideas/Research questions**	**Examples of how it would advance the field of multi-teaming**
**Stream 1**. Social Psychology WITH Management/Applied Psychology	• What are the effects of multi-teaming (both organizational and social/personal sphere) on individual well-being, organizational and team outcomes?	• Establish if multi-teaming is generalizable across settings (e.g., individual characteristics that predict multi-teaming in organizational settings also predict multi-teaming in personal/social settings and the other way around); • Similar to the work-life balance research stream establish whether social groups act as demands/resources for the organizational groups and the other way around; • Examine how multi-teaming in organizational and personal/social settings together contribute to identity formation, emergence of meaning and general life satisfaction. • Facilitate the flow of theories, methodologies and operationalizations from one field to another.
**Stream 2**. Operations Research and Management Science, Industrial Engineering and Computer Science and Artificial Intelligence WITH Management/Applied Psychology	• Integration of the insights stemming from applied psychology and management literature on the consequences of multi-teaming for individual employees into the formal modeling and organizing of multi-teaming.	• Examine which is the optimal number of teams/projects employees can be allocated to; • Explore which is the best way of scheduling activities and tasks shifts for individual employees working on multiple projects. • Examine the effects of scheduling algorithms/heuristics on individual well-being and team satisfaction.
**Stream 3**. Industrial Engineering, and Computer Science and Artificial Intelligence WITH Management/Applied Psychology WITH Social Psychology	• Considering the technological development and the increased usage of virtual work/work from home, digitalization and computerization in relation to work as well as non-work related, social context of individuals.	• To what extent the increased usage of technology (virtual work, work from home) at work affects non-work related social groups (e.g., isolation) and the other way around.

A second main finding is that the intellectual structure of the field is divided in two parts. On the one hand we have the research stream dealing with issues related with multi-project scheduling. On the other hand, we have the stream looking at the antecedents and consequences of multi-teaming, more from an organizational behavior and management perspective. These two streams are poorly connected with each other despite the fact that they are both looking at the same phenomenon of multi-teaming. Given this gap we propose a third stream of research that integrates Operations Research and Management Science, Industrial Engineering and Computer Science and Artificial Intelligence with Management/Applied Psychology. This stream could integrate the insights stemming from applied psychology and management literature on the consequences of multi-teaming for individual employees into the formal modeling and organizing of multi-teaming. Researchers could explore for example which is the optimal number of teams/projects employees can be allocated to or which is the best way of scheduling activities and tasks shifts for individual employees working on multiple projects.

A final future research stream we propose looks at the integration between Industrial Engineering, and Computer Science and Artificial Intelligence with Management/Applied Psychology and Social Psychology. This is an integration across three fields. Within this stream researchers can look at the relation between technological development and the increased usage of virtual work in relation to multi-teaming both in the work as well as the personal sphere. Possible questions here could be to what extent the increased usage of technology (virtual work, work from home) at work affects non-work related social groups (e.g., isolation) and the other way around.

## Limitations

Bibliometrics methods and review studies, as with every study, are not immune to limitations. First, although the keywords chosen are based on a panel of expert scholars in the field and we believe provide face validity, it may be the case that the inclusion or exclusion of some keywords might to a certain degree skew the results (e.g., Batistič and van der Laken, 2019). The same principle applies to the selection of threshold in this study. As Zupic and Čater (2015, p. 13) noticed: “establishing the level of citation thresholds is a part of bibliometric analysis that is definitely more art than science”. To minimize this issue we followed guidelines from previous studies (e.g., Batistič and van der Laken, 2019) and explored the impact of the selection of different thresholds on the results and we did not substantially differentiate. Nonetheless, the authors suggest that regardless of the threshold, the most important documents are included (e.g., due to high citation count), and clusters grouping from one analysis is reflected also in others, acknowledging the most important aspect of providing an accurate and comprehensive visualization of the research field in question (Bunjak et al., 2022).

Second, bibliometric approaches do not capture why authors cite others (Zupic and Čater, 2015). It might be the case that some citations are a consequence of self-legitimization strategies, peer review processes or criticism about specific work (Zupic and Čater, 2015). Such issues are indeed important, but on one hand, some of them, like self-legitimization strategies (e.g., self-citation or citation from friends) cannot be addressed by the bibliometric methods, but on the other hand, some studies suggest that such issues are an organic process of the citation process and as such, they should not be removed from important inferential statistics (Glänzel et al., 2006).

Lastly, once the bibliometric software produces the network map, the authors must carefully interpret the provided clusters and try not to fit their analysis to their existing preconceptions (Zupic and Čater, 2015). If not done objectively the analysis could lose its main advantage—objectivity compared to other review studies. Additionally, some bibliometric software, like in our case VOSviewer, does not provide the possibility to supplement the visualization with calculated network indexes as with standard network analysis. This leaves the interpretation of the cluster to visual inspection and their composition, yet the size, position and related visual elements (e.g., the distance between documents) provide the research with a clear idea of the most impactful studies and clusters (Batistič and van der Laken, 2019; van Eck and Waltman, 2019; Bunjak et al., 2022).

## Conclusion

Multi-teaming is a salient part of how we organize our work and social life. The concept was approached from the perspective of various disciplines while having different labels such as multi-teams, multiple teams, project teams, multiple groups, which lead to a fragmented understanding of the field. In particular, our analyses reveal a clear distinction on studies that focused on performance, learning and well-being consequences of multi-teaming (organization focus) and studies that focus on optimizing the organization of simultaneous multiple projects in organizational settings (organizing focus). Our bibliometric analysis revealed that the different disciplines capturing the concept of multi-teaming are moving from fragmentation toward integration. However, there is still room for multi-disciplinary collaborations that would advance the field of multi-teaming. In particular, we point toward ways in which the insights on the performance and well-being consequences of multi-teaming can be further incorporated into the formal modeling of organizing and scheduling multiple simultaneous projects.

## Data availability statement

The raw data supporting the conclusions of this article will be made available by the authors, without undue reservation.

## Author contributions

Design, data analysis and interpretation, writing and revising the manuscript, and approval for publication: NM, SB, RK, PC, and OF. All authors contributed to the article and approved the submitted version.

## Footnotes

^1^To be noted that bibliometric analysis can be performed at different levels of analysis: document, journal, author, organization, country. In this paper, in line with our research goals we only focus on document level and journal level. This means that the nodes in the network are either documents or journals.

^2^For various bibliometric analyses we used different thresholds in order to capture the most adequate visualizations for each analysis; In general, there are no specific guidelines on what is an adequate threshold for each type of analysis (Zupic and Čater, 2015).
